# GPT-4 passes the bar exam

**DOI:** 10.1098/rsta.2023.0254

**Published:** 2024-04-15

**Authors:** Daniel Martin Katz, Michael James Bommarito, Shang Gao, Pablo Arredondo

**Affiliations:** ^1^ Illinois Tech, Chicago Kent College of Law, Chicago, IL, USA; ^2^ CodeX, The Stanford Center for Legal Informatics, Stanford, CA, USA; ^3^ Bucerius Law School, Hamburg, Germany; ^4^ 273 Ventures, LLC, USA; ^5^ Casetext, Inc., USA

**Keywords:** large language models, Bar Exam, GPT-4, legal services, legal complexity, legal language

## Abstract

In this paper, we experimentally evaluate the zero-shot performance of GPT-4 against prior generations of GPT on the entire uniform bar examination (UBE), including not only the multiple-choice multistate bar examination (MBE), but also the open-ended multistate essay exam (MEE) and multistate performance test (MPT) components. On the MBE, GPT-4 significantly outperforms both human test-takers and prior models, demonstrating a 26% increase over ChatGPT and beating humans in five of seven subject areas. On the MEE and MPT, which have not previously been evaluated by scholars, GPT-4 scores an average of 4.2/6.0 when compared with much lower scores for ChatGPT. Graded across the UBE components, in the manner in which a human test-taker would be, GPT-4 scores approximately 297 points, significantly in excess of the passing threshold for all UBE jurisdictions. These findings document not just the rapid and remarkable advance of large language model performance generally, but also the potential for such models to support the delivery of legal services in society.

This article is part of the theme issue ‘A complexity science approach to law and governance’.

## 1. Introduction

It is difficult to imagine a professional field for which natural language is more integral than the law. As part of their daily activities, legal professionals like judges, regulators, legislators and lawyers spend countless hours consuming and/or producing a wide variety of legal documents. The document types are varied but include legal texts such as statutes, regulations, judicial decisions, contracts, patents, briefs, opinion letters, memos and other related materials [1,2].

Legal language is notoriously complex [3–5], and the ability to interpret such complex documents often requires years of study. Indeed, part of the charge of legal education is, in fact, a linguistic immersion program where students are trained to parse both the syntactic and semantic nuances of various legal texts [6,7]. There are many sources of complexity in legal language: for example, words like ‘security’ that have common meaning in normal language often have different, context-specific meanings in legal language. Many words that do not occur at all in normal language, like ‘estoppel’ or ‘indemnitor,’ occur regularly in legal corpora [8]. This semantic depth and breadth is challenging for those not otherwise familiar with the legal lexicon. The public, for example, is quite aware of the linguistic gap between general language and legal language, referred to by many as legalese [9–11].

The complexity of the law [12–15] imposes real consequences for many individuals and organizations [16,17]. In part due to complexity, legal systems have struggled to assist with the quantity, quality, and accessibility of legal services demanded by society [17–19]. A technology-based force multiplier [19,20] is arguably needed to help support the high cost and unmet demand for legal services [21,22]. Yet, in order for technology systems to meet this need, they must confront the nuances of legal languages and the difficulties of complex legal reasoning tasks [23]. Unfortunately, from a historical perspective, computational technologies have struggled not only with natural language processing (NLP) tasks generally, but, in particular, with complex or domain-specific tasks like those in law.

There is promise on the horizon, however; state-of-the-art performance in NLP has advanced substantially over the last decade, largely driven by advances in computer hardware, data availability and neural techniques. Indeed, cutting-edge work within the field of NLP has recently undergone a rapid transition where classical NLP methods have been supplanted by neural based methods [24,25]. While neural techniques have a long history [26–29], current modelling approaches generally trace their lineage to the arc from shallow embeddings trained on CPUs to the current transformer-based architectures optimized for purpose-built, distributed GPU/TPU infrastructure [30–41].

While there is an increasing number of generally accessible large language models (LLMs), the best known of these are from OpenAI’s family of Generative Pre-trained Transformer models, commonly referred to as GPT [38,42–45]. In November 2022, OpenAI released a chat interface to a version of its ‘GPT-3.5’ models, colloquially known as ChatGPT, which reportedly resulted in millions of sign-ups within days of release and over 100M users in the first 100 days [46]. As described by OpenAI, GPT-4 is ‘a transformer-style model pre-trained to predict the next token in a document, using both publicly available data (such as internet data) and data licensed from third-party providers. The model was then fine-tuned using reinforcement learning from human feedback (RLHF)’ [42]. While this family of models encompasses a range of tasks, sizes, and training techniques and continues to expand, all models are generally trained using reinforcement learning or supervised fine-tuning on billions of tokens and parameters.

NLP models have progressed in the legal domain [23,47,48] with increasing application of neural techniques on specific legal tasks [49–51]. Several recent papers have demonstrated meaningful zero-shot progress on a variety of applied tasks [2,52–56], suggesting further potential for application as the state of the art improves.

Recognizing the advancing capabilities of large language models, we sought an exemplary challenge to demonstrate this potential to both the legal domain and general scientific community. While models such as GPT-2 have shown promising results at parsing syntax [57,58], some have argued against the possibility that a language model could exhibit complex semantic reasoning [59–61]. However, in recent prior work [8], a subset of the authors demonstrated the near-passing zero-shot performance of text-davinci-003 on the multiple choice component (MBE) of the uniform bar exam (UBE)—a task which requires both extensive domain knowledge and a significant degree of semantic and syntactic command of the English language. While no prompts or parameters met a ‘passing’ level, the rate of performance increase from text-davinci-001 to text-davinci-003 strongly suggested that passing performance could ‘occur within the next 0–18 months’ [8]. In this paper, we demonstrate that this time has come for not only the MBE, but also the essay (MEE) and performance test (MPT) components of the UBE. As demonstrated by the zero-shot performance results we report herein, GPT-4 can ‘pass the Bar’ in all UBE jurisdictions.

## 2. The Uniform Bar Exam

### (a) Description of the Uniform Bar Exam

The vast majority of jurisdictions in the USA require the completion of a professional licensure exam (the bar exam) as a precondition to practice law. The bar exam is a challenging battery of tests arguably designed to evaluate an applicant’s legal knowledge and skills. Successfully passing the Exam requires that an examinee display some degree of ability to discern challenging factual and legal scenarios, understand and apply legal principles, and both consume and produce complex legal language.

In order to sit for the exam, the typical applicant must complete at least seven years of post-secondary education, including completion of a 4-year undergraduate degree, followed by matriculation and graduation from a law school accredited by the American Bar Association. In addition to these years of education, most applicants also invest substantial amounts of time and money into specialized test-taking courses [62]. Despite this effort and investment, roughly one in five test-takers is unable to pass the Exam on their first attempt.

Attorney licensure is a topic governed by the states, typically through rules promulgated at the direction of state supreme courts [63]. Thus, each state is responsible for selecting its own requirements and methods of exam administration. Notwithstanding such broad authority, many states have selected to standardize their requirements. Over the past decade, more jurisdictions have chosen to participate in the UBE [62,64]. Despite this push toward greater uniformity, however, there are often additional requirements, even within states that have adopted the UBE, such as the multistate professional responsibility examination (MPRE) or state-specific subject matter areas. In this paper, we address only the UBE as produced by the National Conference of Bar Examiners (NCBE). The core UBE components, outlined in table 1 below, are the multistate bar exam (MBE), the multistate essay exam (MEE) and multistate performance test (MPT).

**Table 1.** Summary of uniform bar exam (UBE) components.

UBE component	total UBE points	questions	time	time per question
multistate bar exam (MBE)	200 points	200 questions	6 h	1 min 48 s
		(multiple choice)		
multistate essay exam (MEE)	120 points	6 questions	3 h	30 min
		(3–4 subquestions)		
multistate performance test (MPT)	80 points	2 questions	3 h	90 min
		(3–4 subquestions)		

As shown in table 8 and discussed in detail in the electronic supplementary material, the UBE is a 12 hour exam taken over 2 days, with the MPT and MEE administered on Day 1 while the MBE is administered on Day 2. The UBE is scored on a total scale of 400 points, with the scores from all three sections scored together. In general, there are no minimums required for a specific component of the exam, as a strong score on one component can help an examinee overcome a weaker score on another component. As displayed in the electronic supplementary material, a combined score of 266 points is enough to pass in jurisdictions such as Illinois, New York and the District of Columbia, while a score of 270 points would pass in the vast majority of states which use the UBE.

## 3. Data and methods

### (a) Data

The primary focus of the NCBE is on the construction of exams for use on a nationwide basis. The NCBE exams are developed in an institutional context by the organization’s staff and advisors, who have many years of experience designing, scoring and calibrating these exams across US jurisdictions.

As noted earlier, the UBE has three separate components: the MBE, MEE and the MPT. In order to analyse whether GPT-4 could pass the Bar Exam, we collected relevant materials for each of the three separate UBE components. For the MEE and the MPT, we collected the most recently released questions from the July 2022 Bar Examination. These questions are readily available through the websites of many state bars. The July 2022 MEE exam features six questions, covering Evidence, Contracts, Corporations, Trusts, Civil Procedure and Real Property. The two questions for the July 2022 MPT required test-takers to (i) draft a memo in the context of a domestic relations matter with a series of choice of law issues and (ii) construct an objective memo focused on questions of criminal law and legal ethics.

The MBE questions used in this study are official multistate bar examination questions from previous administrations of the UBE [65]. The MBE full-length exam we use is subject-weighted in near-equal proportion across the seven core subject matter areas. While the exact sequence of questions administered is not identical to any actual exam as administered, it has been described by the NCBE itself as ‘the first [MBE Complete Practice Exam] from NCBE to mimic a full-length MBE.’ [65] While we are not able to release the MBE questions, the questions can be purchased directly from an NCBE authorized reseller.

Links to access both the full length MEE and MPT questions, as well as their representative ‘good’ answers, are available in the online Github repository (https://github.com/mjbommar/gpt4-passes-the-bar). These representative good answers are made available by state bar associations and reflect actual MEE and MPT answers produced by real examinees. These answers are described as neither ‘average passing answers nor are they necessarily perfect answers.’ We would suggest that the interested reader review these representative ‘good’ answers side-by-side with our model outputs.^1^

### (b) Methods

Given the sheer size of the training data used to develop GPT-4, as a threshold matter, we needed to ensure that none of the test data had somehow leaked into the training set. We were able to work directly with OpenAI to run a contamination check on each of the questions we leverage herein. Based on the analysis conducted by OpenAI and reported in table 9 of the GPT-4 technical report, the questions we processed within our test set are not within the provenance of model [42].

In prior work, a subset of the authors implemented and described frameworks for multiple-choice assessment on the Bar Exam [8] and an open-ended assessment for task-based simulation in the CPA Exam [66]. We follow the approach outlined in prior work to the extent possible, including input-formatting conventions, prompt styles and model parameters.

#### (i) MBE

As described above, the MBE section is administered as a multiple-choice question exam. For each session, in which a model sits for a complete exam, each question is sent to the model with a formatted prompt. All model responses are logged, including all available API metadata, and stored for subsequent review. All grading is automated. Experiments involved three runs for each set of prompts and parameters; we report the average score across all runs in our results. Additional details are available in the online repository (https://github.com/mjbommar/gpt4-passes-the-bar).

#### (ii) MEE and MPT

Unlike the MBE, both the MEE and MPT are administered as open-ended exams. These exams combine background and reference material with one or more sub-questions in a manner similar to the task-based simulations in other professional licensing examinations. We standardize the formatting of these materials and questions to generate plain text versions of the Exam. For each session, each question is sent to the model and its response is logged for subsequent grading and review. Additional details are available in the online repository (https://github.com/mjbommar/gpt4-passes-the-bar).

## 4. Results

### (a) Multistate bar exam results

#### (i) Overall MBE results

**Figure 1.**
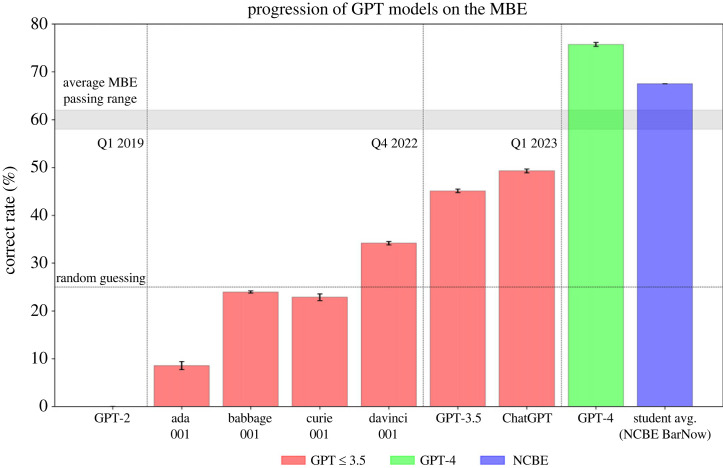
Progression of recent GPT models on the multistate bar exam (MBE). (Online version in colour.)

We administered the MBE to most available GPT models, including not only GPT-4, but also an alpha version of ChatGPT, text-davinci-003, text-davinci-001, text-curie-001, text-babbage-001 and text-ada-001. The per-model accuracy averaged across all model runs is presented in table 2 and visualized in figure 1.

**Table 2.** Accuracy of GPT Models on the multistate bar exam (MBE).

model name	MBE accuracy
GPT-4	75.7%
chat-davinci-003-alpha	49.2%
text-davinci-003	45.1%
text-davinci-001	34.2%
text-babbage-001	23.9%
text-curie-001	22.8%
text-ada-001	8.5%
GPT-2	N/A

Table 2 demonstrates the increase in MBE performance between even the most recent members of the GPT family and the alpha version of GPT-4 we used for this study. GPT-4 delivers a 26.5% increase in the accuracy over ChatGPT, the previously best performing model. In addition, GPT-4’s MBE score is not only more than 15% above the minimum passing threshold, but also outperforms the average human test-taker by more than 7%.

Table 2 and figure 1 highlight the broader progression of GPT models since 2019. Some of the earliest models such as GPT-2 [44] are unable to process prompts consistently, while later models such as Curie (text-curie-001), Babbage (text-babbage-001) and Ada (text-ada-001) were unable to obtain performance above that of the statistical guessing rate of 25%. GPT-3 (text-davinci-001) (initially released in 2020) [38] was the first model to consistently outperform statistical chance. The previously best-available models, ChatGPT (chat-davinci-003) and GPT-3.5 (text-davinci-003), performed just under the 50% accuracy level. As displayed in figure 1, the benchmarked growth on this task is reminiscent of similar nonlinear improvements witnessed within other recent benchmarks [67–69], where over the course of a relatively short period of time leading language models were able to surpass the performance of experts on previously untouchable tasks [70].

#### (ii) MBE results by legal subject area

While GPT-4’s performance on the MBE exceeds the passing rate and the performance of the average human test-taker, it is also interesting to explore its performance within individual legal subjects. Human test-takers perform differentially across the various topics within the UBE. The NCBE Bar Now Platform maintains statistical information regarding student average performance by topic within MBE questions. Table 3 offers both the NCBE Bar Now average accuracy by question category as well as the overall approximate national average MBE performance for recent test-takers. Among other things, table 3 highlights the nature of the challenge which the MBE presents to test-takers as the average student answers more than three in ten questions incorrectly.

**Table 3.** Summary of performance by legal subject area.

legal subject area	GPT-4	ChatGPT	GPT-3.5	NCBE student avg.
civil procedure	**61.1%**	34.9%	39.1%	59%
constitutional law	69.4%	54.2%	45.9%	**72%**
contracts	**88.1%**	48.5%	37.0%	70%
criminal law and procedure	**81.1%**	46.3%	49.8%	71%
evidence	**85.2%**	49.8%	45.7%	65%
real property	**79.7%**	67.8%	52.9%	65%
torts	64.9%	43.2%	45.7%	**71%**
**average accuracy**	**75.7%**	49.2%	45.1%	68.0%

Whether it involves a subject matter expert or a model, there are subjects where performance will likely differ, and, unsurprisingly, some subjects may require more knowledge or be more complex. All of this is conditioned on prior exposure to a topic (data), the nature of that exposure (feedback) and the ability to weight those prior exposures (calibration). While our understanding of LLMs is still nascent [71–73] and we do not fully understand why GPT-4 performs differently by substantive topic, it is likely that the prospect of future performance improvements will, in part, hinge on identifying opportunities for additional topically relevant data, feedback and calibration.^2^

**Figure 2.**
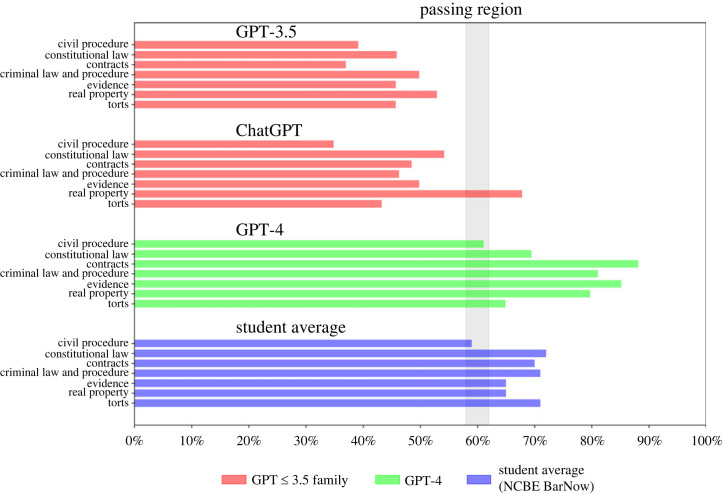
Progression of recent GPT models by legal subject area. (Online version in colour.)

In table 3, we report the MBE results by legal subject area and visualize those same results in figure 2. Both the table and figure reveal that, while GPT-4’s performance may vary by subject, it meets or exceeds the approximate passing threshold in all seven subject matter areas. In addition, it outperforms the average NCBE BarNow test-taker in five out of seven categories (*Civil Procedure*, *Contracts*, *Criminal Law and Procedure*, *Evidence* and *Real Property*).

GPT-4 is now Pareto dominant over all prior versions of GPT (sometimes by a very large margin). *Contracts* and *Evidence* are the topics with the largest overall improvement. GPT-4 achieves a nearly 40% increase over ChatGPT in *Contracts* and a more than 35% raw increase in *Evidence*. *Civil Procedure* is both the worst subject for GPT-4, ChatGPT and human test-takers. However, *Civil Procedure* is a topic where GPT-4 was able to generate 26% raw increase over ChatGPT. This increase pushed GPT-4 into both the passing threshold and beyond the performance of the average NCBE BarNow test-taker.

An observant reader may note that the rank ordering of subject matter performance for text-davinci-003 on this MBE Exam differs from the MBE Exam tested in prior work [8]. Several factors could explain these differences. First, it is important to note that headline 50.3% result in prior work reflects best prompt and parameter, not the averages calculated across all prompts and parameters for text-davinci-003; therefore, the reader should compare these figures to the 42–46% accuracy reported in the prior work’s tables. Second, in this research, we use the most recently published MBE Exam, which the NCBE describes as ‘the first from NCBE to mimic a full-length MBE.’ Differences in the distribution of questions, the distribution of question difficulty or the design of the Exam over time may account for additional variation.

#### (iii) Non-entailment MBE results

Building up the broader concept of textual entailment [74–76], earlier work studying Bar Exams treated ‘the relationship between the question and the multiple-choice answers as a form of textual entailment’ [77] where the ability to identify wrong answers (non-entailment) is differentiated from the ability to identify the correct answer (entailment). Intuitively, this is related to the classic test taking strategy of eliminating clearly erroneous answers. While earlier models are able to undertake this elimination task to a fairly reasonable extent, it is with regard to this non-entailment task that GPT-4 shows its particular strength.

Table 4 reproduces the model accuracy (entailment) and reports the ‘Top 2’ and ‘Top 3’ accuracy (non-entailment). Across the various topics, GPT-4 generally achieves strong ‘Top 2’ performance (i.e. ability to reduce the number of likely answers from four to two). GPT-4’s performance on the ‘Top 2’ task is roughly in line with the ‘Top 3’ performance of prior models. For example, for the *Contracts*-related problems, GPT-4 is able to identify the right answer within its ‘Top 2’ choices in nearly 97% of instances. By contrast, for *Torts*, in roughly 15% of instances, GPT-4 is unable to eliminate even a single wrong answer (which is to say it actually ranks the correct answer as least likely to be correct). This entailment versus non-entailment perspective is one way to consider potential sources of future model improvement. Overall, GPT-4’s second best answer is highly correlated with correctness for all subjects other than *Criminal Law and Procedure*, and its overall performance in rank-ordering responses demonstrates state-of-the-art capabilities for information retrieval tasks.

**Table 4.** Summary of non-entailment performance by legal subject area.

	GPT-4	GPT-4	GPT-4	ChatGPT	ChatGPT	ChatGPT	GPT-3.5	GPT-3.5	GPT-3.5
model	accuracy	Top 2	Top 3	accuracy	Top 2	Top 3	accuracy	Top 2	Top 3
civil procedure	**61.1%**	**82.2%**	**95.4%**	34.9%	55.4%	89.3%	39.1%	49.6%	76.1%
constitutional law	**69.4%**	**86.3%**	**98.6%**	54.2%	73.2%	93.5%	45.9%	59.8%	83.7%
contracts	**88.1%**	**96.7%**	**99.8%**	48.5%	65.5%	88.9%	37.0%	59.8%	78.0%
criminal law and procedure	**81.1%**	**84.6%**	**97.3%**	46.3%	77.8%	92.8%	49.8%	63.9%	77.7%
evidence	**85.2%**	**91.5%**	**99.8%**	49.8%	69.8%	89.6%	45.7%	60.3%	80.7%
real property	**79.7%**	**87.9%**	**100.0%**	67.8%	76.2%	94.4%	52.9%	69.0%	78.0%
torts	**64.9%**	**73.6%**	**85.5%**	43.2%	55.6%	73.0%	45.7%	58.4%	66.8%

### (b) Multistate essay examination results

Many would consider the construction of essays to be a more difficult task than answering multiple choice questions, particularly for a computational system. While selecting an answer from a difficult but otherwise already defined list of choices is certainly a challenge, it is arguably a much more challenging task to read and identify key issues in a one page prompt and then draft a fulsome essay on a complex subject matter.

We experimented with a variety of prompts, hyperparameter settings and question formatting techniques. Among other things, this initial analysis revealed the clear benefit of question segmentation. Namely, both GPT-4 and ChatGPT delivered more detailed results when both were provided with a single MEE subquestion to consider. Thus, for each MEE question, we made one small modification from the problem as presented. We ran each MEE subquestion one at a time and lightly corrected the language so as to craft the question in the form of a complete sentence. As an example, consider the questions posed in figure 5, electronic supplementary material. Then, imagine them delivered together with the vignette one question at a time to each of the models. Access to the prompt as administered is available the online repository (https://github.com/mjbommar/gpt4-passes-the-bar).

Two of this study’s authors, a tenured law professor and an attorney licensed in multiple jurisdictions, reviewed the model output and collaboratively assigned scores to each of the MEE questions. Understanding we could not directly replicate the process followed by the NCBE, we debated each score with the view that we should be somewhat conservative in the assignment of MEE scores. As an additional check, we also solicited analysis from peers who were provided with selected samples of the model’s responses and reached assessments that met or exceeded our own. While we recognize there is inherent variability in any qualitative assessment, our reliance on the state bars’ representative ‘good’ answers, our internal debates and conservative standard as well as the use of other individuals reduces the likelihood that our assessment is incorrect enough to alter the ultimate conclusions set forth in this paper.

In figures 6–12 (located in the Electronic Supplement), we reproduce output for the July 2022 MEE Evidence Question for three models (GPT-4, ChatGPT and GPT-3.0). Although the primary focus of our analysis is the comparison between more recent GPT models, in figure 12, we reproduce the GPT-3.0 MEE model’s answer for the overall evidence question. Similar to the trend previously shown in figure 1, we believe that observing the broader progression across these models helps highlight the underlying increase in capabilities. The pattern revealed in figure 1 is also true for the MEE and the side-by-side comparison of output should help the interested reader observe this arc. In addition, the side-by-side comparison of the model output from the MEE Evidence question also reveals some of the broader patterns reflected across the balance of the MEE essay problems.

Starting with the oldest model first, GPT-3.0 produces very thin output in response to a prompt which specifically directs it to produce a fulsome answer. As shown in figure 12, GPT-3.0 can vaguely recite some of the relevant principles and rules but does not properly connect those principles to the facts and consistently reaches improper legal conclusions. In the context of this complex legal problem, GPT-3.0 is far below the mark.

While the weaknesses in the GPT-3.0 output are clear, the comparison between GPT-4 and ChatGPT requires a more nuanced analysis. In figures 6 and 9, we reproduce the output for the first subquestion within the July 2022 MEE Evidence problem. An initial review of that output reveals that ChatGPT actually produces a slightly longer response than GPT-4. At a substantive level, however, and particularly as compared to GPT-4, ChatGPT is deficient in several important ways. Unlike GPT-4, ChatGPT fails to properly identify all four prongs of Rule 702 of the Federal Rules of Evidence (FRE). This results in a failure to discuss Rule 702(a). Yet, under FRE Rule 702, all four prongs (including 702 (a)) must be satisfied in order for the expert’s testimony to be allowed. In addition to missing this important discussion, ChatGPT begins to meander intellectually and provides an analysis of FRE Rule 403. While not totally unrelated, this is a rule which is out of scope for this question. By contrast, GPT-4 does an overall better job of addressing the question presented by properly citing the relevant law, connecting the law to the facts and otherwise staying on topic.

This basic dynamic is replicated across not only the balance of the MEE Evidence question but also across much of the MEE model output. As an additional example, consider the second subquestion on the MEE Evidence problem (figures 7 and 10) where ChatGPT fails to address a relevant rule (FRE 403) and instead focuses significant attention on non-relevant rules (FRE 701 and 702). It is arguably not proper to call this a model hallucination in the sense that these are real rules which are, in fact, somewhat related to the question. These topics are simply out of scope with respect to the specifics of the question that was posed. GPT-4, by contrast, correctly discusses both FRE Rule 403 and Rule 404(b) and does not devote attention to extraneous issues.

While GPT-4 performs well on many questions, its output is not completely free of errors. In the three sub-questions that we assign the lowest scores, GPT-4 produces several notable errors. First, it has difficulty calculating the distribution of assets from a testamentary trust which has been deemed to be invalid. Next, it fails to grasp the call of the question and provides an incorrect answer on a civil procedure question regarding diversity jurisdiction after the joinder of a necessary party. Finally, GPT-4 provides improper analysis on a real property (real estate) subquestion regarding both the proper designation of a Future Interest and the application of the Rule Against Perpetuities.

It should be noted that several of these topics where GPT-4 struggles are also areas where law students and bar examinees would also likely struggle. In particular, the Rule Against Perpetuities is considered by many to be among the most difficult issues in all of law. In addition, it should be mentioned that most real life examinees who otherwise pass the Bar Exam are unable to complete an end-to-end MEE that is free from errors. Overall, even in problems for which we assign a lower grade, GPT-4 is often able to deliver a partial answer, such as identifying some, but not all, relevant legal principles or providing reasonable discussion of some of the facts that are relevant to the legal question.

Due to space constraints, we reproduced only the first MEE question (i.e. July 2022 MEE Question 1) and output within the electronic supplementary material. However, we would once again direct the interested reader to the online repository (https://github.com/mjbommar/gpt4-passes-the-bar), which features all model outputs for each of the July 2022 MEE questions, our assigned subquestion grades, links to representative ‘good’ answers and other useful information. Overall, as presented in table 5, our final MEE grade is 4.2 out of 6 points for GPT-4 and 3 out of 6 points for ChatGPT. Most jurisdictions leverage the six-point scale, where a score of four or higher is generally considered passing. While we believe the output for GPT-4 compares favorably when compared with the representative good answers, as noted earlier, we were unable to replicate a full scale grading process identical to that undertaken by the NCBE in the actual administration of the bar exam. Thus, we encourage the interested reader to review all model output and to reach their own conclusions regarding the quality of the MEE answers produced by the respective models.

**Table 5.** Summary of performance by multistate essay examination (MEE) question category.

MEE question subject	GPT-4	ChatGPT
MEE 1 - evidence	5.0/6.0	3.7/6.0
MEE 2 - contracts	4.2/6.0	3.1/6.0
MEE 3 - corporations	4.4/6.0	3.0/6.0
MEE 4 - trusts/estates	3.9/6.0	2.5/6.0
MEE 5 - civil procedure	3.5/6.0	2.8/6.0
MEE 6 - real property	4.2/6.0	2.7/6.0
**overall score**	4.2	3.0

### (c) Multistate performance test results

As discussed earlier, the July 2022 MPT features two substantive problems: one question focused upon a complex family law matter with embedded choice of law issues and another question focused on a mixture of criminal law and legal ethics issues. For purposes of comparative analysis, we evaluate the performance of GPT-4 relative to earlier models, such as ChatGPT. As many have noted, prior models were unable to handle longer documents in zero-shot tasks due to their token limits. The July 2022 MPT featured two questions, each over the publicly available ChatGPT 4096 token limit—5297 tokens for MPT-1 and 5188 tokens for MPT-2. However, thanks to assistance from OpenAI, we were able to use an ‘8K’ version of ChatGPT that has a wider context window of 8193 tokens, and could thus accommodate the length of the overall materials. This longer context window was critical to our analysis.

Similar to the question-by-question approach that we undertook for the MEE, for both MPT-1 and MPT-2, we presented the problems as subquestions. First, we placed the instructional memo (describing the task to be undertaken) at the end of the prompt (after ‘the File’ and ‘the Library’). Second, we reduced the memo to a single subquestion for each prompt. Thus, for MPT-1 we ran the model four times, one time per subquestion.

Both of the models we considered produced long-form answers to the respective problems, but there are some fairly straightforward differences in the quality of output produced. For purposes of analysis and discussion, we will focus upon the July 2022 MPT-1 problem. Figure 13 (located in the Electronic Supplement) replicates the MPT-1 instructional memo while figures 14–21 (located in the Electronic Supplement) reproduce the MPT-1 model output from both GPT-4 and ChatGPT.

Following an approach comparable to our process for the MEE, we reviewed the MPT output produced by each model and evaluated it against the representative ‘good’ answers. We then assigned scores to both of the MPT questions from the July 2022 exam. These results are reported in table 6. As we noted in the grading of the MEE, we recognize that there is a subjective aspect to this sort of analysis and so we encourage the interested reader to review the MPT output contained in the Electronic Supplement as well as the output from MPT-2 in the online repository (https://github.com/mjbommar/gpt4-passes-the-bar) and reach their own conclusions.

**Table 6.** Summary of performance on multistate performance test (MPT) questions.

MPT question	GPT-4	ChatGPT
MPT 1 - Hixon Marriages	4.2/6.0	3.0/6.0
MPT 2 - In re Briotti	4.1/6.0	2.5/6.0
**overall score**	4.2	2.8

Beyond the numeric results displayed in table 6, our grading procedure revealed some clear and important distinctions in the quality of the output as between GPT-4 and ChatGPT. For example, in the first subquestion of MPT-1, both GPT-4 and ChatGPT produce a lengthy and otherwise reasonable looking answer. Similar to differences in the MEE scores, the differences in quality manifest themselves within the deeper details of the problem. In figure 18, ChatGPT correctly identifies that it should address the Restatement of the Conflicts of Law as this is core to the answer. However, it incorrectly cites §6 as the proper source when it should cite §283 as the source of the ‘significant relationship’ test.^3^ More fundamentally, despite partial success on the problem, ChatGPT ultimately draws the incorrect legal conclusion. Columbia law and not Franklin law will likely govern the question of annulment. In figure 14, GPT-4 not only correctly identifies that Columbia Law is controlling but provides reasonable arguments as to why Columbia should be selected under the ‘significant relationship’ test.

Subquestion three of MPT-1, as displayed in figures 16 and 20, provides another example of the distinction in quality between GPT-4 and ChatGPT. Here, GPT-4 correctly distinguishes between the ability of the Franklin court to annul the marriage and its inability to dispose of the parties’ property. The Franklin Court cannot, without personal jurisdiction over Tucker, take action against a property outside its borders. Ms. Tucker is not a resident of Franklin and the property is not in Franklin. Her source of contact with Franklin is limited to the fact that her soon to be ex-husband moved there. ChatGPT fails to distinguish between the circumstances and assigns authority to the Franklin Courts that they do not possess. These type of patterns repeat themselves over the remainder of both MPT-1 and MPT-2.

It was our hypothesis that the MPT would prove to be more challenging to GPT-4 than the MEE. While the MEE requires the examinee to answer substantive law questions from any of the potential topics within the list of bar exam subjects, the MPT is a somewhat different type of exercise. It is a lawyering exercise where the materials as provided define the relevant universe of information. As the NCBE describes it, ‘the MPT is not a test of substantive law; the Library materials provide sufficient substantive information to complete the task.’ Consequently, the MPT requires a suspension of knowledge, whereby the examinee must, for the period of the test, imagine themselves in a jurisdiction that may contradict their actual knowledge of real law. Indeed, the instructions provided with the test remind the test-taker that even ‘if the cases appear familiar to you, do not assume that they are precisely the same as you have read before. Read them thoroughly, as if they all were new to you.’

We were concerned that this ‘suspension of a broader knowledge,’ or ability to work within the four corners of the exam material, would prove challenging for any member of the GPT family (even GPT-4). Thus, we were somewhat surprised at the quality of the output which was generated. Both GPT-4 and, to a lesser extent, ChatGPT were able to largely avoid the trap of citing legal principles, cases, or other materials which would otherwise appear to be on point but would not be responsive to the requirements of the MPT.

### (d) Combined results and comparison to the UBE passing threshold

The UBE has three components (MBE, MEE and MPT) which are typically, but not always, weighed using the approach highlighted in table 1. As displayed in table 8, different jurisdictions impose different UBE passing score thresholds ranging from 260 (for states such as Alabama and Minnesota) to 273 points (for Arizona). The vast majority of UBE states impose a minimum UBE passing score threshold between 260 and 270.

In table 7, we combine all the analysis conducted above to offer an overall UBE score for both GPT-4 and ChatGPT. GPT-4 obtains an overall UBE score of 297 points^4^ while ChatGPT obtains a score of 213 points.^5^ Although GPT-4 obtains within-category passing-level scores for both the MEE and MPT, its high MBE percentile provided it with substantial latitude; GPT-4 would likely pass even with a much lower MEE or MPT score in some or all jurisdictions. In total, our analysis highlights that GPT-4 has indeed passed the Bar and has done so by a significant margin.

**Table 7.** Summary of overall performance on uniform bar exam (UBE).

UBE component	GPT-4	ChatGPT
multistate bar exam (MBE)	157 points	116 points
multistate essay exam (MEE)	84 points	60 points
multistate performance test (MPT)	56 points	37 points
**overall score**	297 points	213 points

## 5. Conclusion

In this paper, we evaluate GPT-4’s zero-shot performance on the entire UBE. The exam, which includes both multiple-choice and open-ended tasks testing theoretical knowledge and practical lawyering, is something that many might consider insurmountable for a computational system. While this paper is designed to merely evaluate the lower end of the technical continuum (e.g. GPT-4 with minimal prompting), the results reported in the paper highlight many other fruitful avenues for future research. Namely, it is almost certainly the case that more exhaustive prompt engineering, few shot and/or other more systematic engineering techniques layered upon the base capabilities of GPT-4 will yield stronger performance than the results we report in this paper. Thus, there is significant opportunity to advance the performance of large language models through techniques such as external queries, scratchpads, chain-of-thought prompting, retrieval augmented generation or one of the many other techniques [79–84].

While we are limiting our focus to GPT-4 for the purposes of this paper, we believe there are several long-term questions for the field including whether other new foundational models (e.g. Gemini, Claude 2), a domain specific legal model (e.g. a LawGPT) or a mixture of models (e.g. K-LLMs) will be able to outperform GPT-4 on not only the Bar Exam but also a range of real life lawyering tasks. Relatedly, will open source models (e.g. Llama 2, Mixtral 8x7B etc.) be able to match or exceed the performance of their closed source counterparts? These are some of the future questions which are likely to be evaluated within the literature in the near term future.

As the demand for better, faster and more affordable legal services is only increasing in society, the need for supporting technology is becoming more acute. Further research on translating the capabilities of LLMs like GPT-4 into real public and private applications will be critical for safe and efficient use [85]. GPT-4, like prior models, may still hallucinate sources, incorrectly interpret facts or fail to follow ethical requirements; for the foreseeable future, applications should feature ‘human-in-the-loop’ workflows or similar safeguards.^6^ However, it appears that the long-awaited legal force multiplier is finally here.

## Data Availability

Output Data are available via our online repository. Access to a same or significantly similar version of the GPT-4 model is generally available under commercial terms. Electronic supplementary material, source code and data are available online at https://github.com/mjbommar/gpt4-passes-the-bar. Supplementary material is available online [86].

## Footnotes

1 When compared with the exam rubrics which offer an exhaustive list of potential topics that an examinee might theoretically produce given unlimited time, the representative good answers reflect exemplary answers selected by bar examiners that were actually produced within the allotted 30 min.

2 We expect that future researchers will delve further into the question of why GPT-4 performs differently by substantive topic.

3 It is challenging because §283 actually does in part invoke §6 but it is not the source of the ‘significant relationship’ test. The sentence as drafted by ChatGPT contains a circular reference (is an infinite loop).

4 Best prompt and/or hyperparameter combination on the MBE would push this score to 298 or higher. Here, we report the MBE average of 75.7% which composites to a 297.

5 For the reader who is not familiar with these issues, it might be difficult to contextualize these UBE scores. Unfortunately, there is no publicly available July 2022 national bar exam percentiles against which to compare these results. However, using a percentile chart from a February 2018 exam administration (which is generally available online), ChatGPT would receive a score below the 10th percentile of test-takers while GPT-4 would receive a combined score approaching the 90th percentile of test-takers. However, it should be noted that this chart might not be the best approach to the estimation given the skew towards ‘retakers’ in the February exam administration [78]. While we are not fully convinced of the methodological approach taken in some subsequent analysis [78], we do agree that it would be better to consider the raw 297 UBE as falling within a range between 68th and 90th percentile (depending on the precise state and timing of the exam administration). See table 8, electronic supplementary material, for additional information.

6 As noted in the GPT-4 Technical Report, ‘GPT-4 has various biases in its outputs that we have taken efforts to correct but which will take some time to fully characterize and manage’ [42].
